# Effects of nutrition education via a mobile-based game on nutritional knowledge, dietary habits and anthropometric indices in preschool children: A study protocol

**DOI:** 10.1016/j.mex.2023.102104

**Published:** 2023-03-02

**Authors:** Maryamsadat Riasatian, Zahra Karimian, Mohammad Ali Mohsenpour, Shiva Faghih

**Affiliations:** a Department of community nutrition, School of Nutrition and Food Sciences, Shiraz University of Medical Sciences, Shiraz, Iran; b Department of e-Learning in Medical Sciences, Virtual School and Center of Excellence in e-learning, Shiraz University of Medical Sciences, Shiraz, Iran; c Department of Clinical Nutrition, School of Nutrition and Food Sciences, Shiraz University of Medical Sciences, Shiraz, Iran

**Keywords:** Gamification, Children's nutrition, m-Health, Dietary diversity score, A study protocol

## Abstract

Preschool age is a great time to learn a healthy lifestyle, for behavior therapy. Mobile health procedures are inexpensive, reliable, and accessible. This project has two phases. The KidFood mobile game and two nutrition knowledge questionnaires were designed during the first phase. In the second phase, a six-month, parallel, blinded, randomized controlled trial will perform on 120 Iranian children aged 5 to 6 years. Before and after nutritional education via KidFood, the dietary habits, the nutritional knowledge of parents and children, and the anthropometric indices of children will be evaluated.

## Graphical abstract

**Figure:**
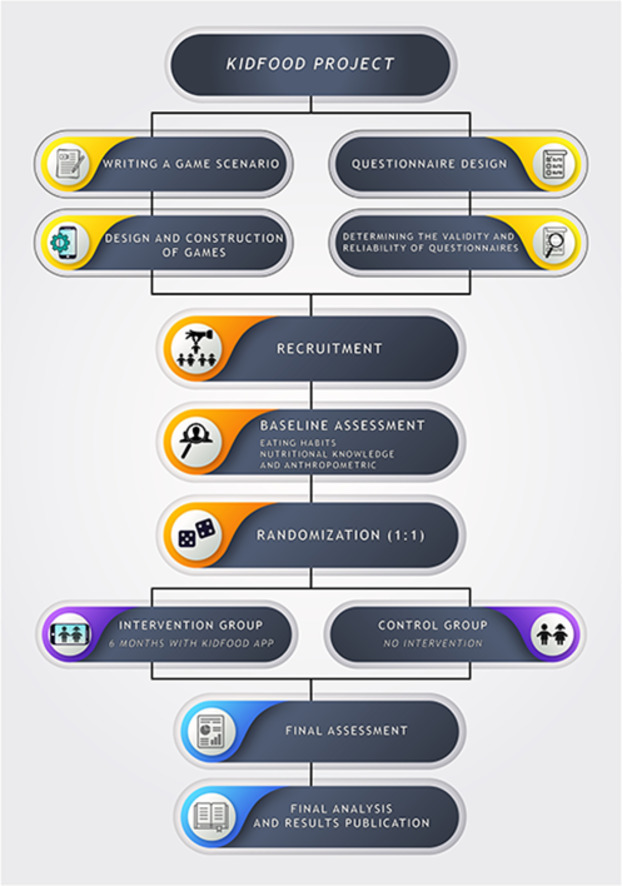

Specifications table

**Table:** 

Subject area:	Medicine and Dentistry
More specific subject area:	Nutrition education for children
Name of your protocol:	Effects of Nutrition Education via a Mobile-Based Game on Nutritional Knowledge, Dietary Habits and Anthropometric Indices in Preschool Children: a study protocol
Reagents/tools:	Kidfood game made by Mahad Knowledge-based institute of Game Development and Animation Nutritionist IV software IBM SPSS Statistics for Windows, version 23.0; IBM Corp
Experimental design:	Nothing
Trial registration:	Iranian Registry of Clinical Trials (IRCT.ir) registry code of IRCT20190810044496N1
Ethics:	• The protocol follows Helsinki Declaration • The relevant informed consent was obtained from parents or guardians • The ethics committee of Shiraz University of Medical Sciences (SUMS), Shiraz, Iran (IR.SUMS.REC.1398.332),
Value of the Protocol:	• Using the learning theory of Jean Piaget, one of the famous constructivists to design education with a game, a New and unique protocol for the mobile-based game in preschoolers. • Explaining the scenario of an educational game. • Designing nutritional knowledge questionnaires for parents and visual children's questionnaires with graphic elements of the game for children following game education instead of using prepared questionnaires.

## Description of protocol

### Background/introduction

Children are ready to learn and accept new experiences [1]. Behavioural treatment to increase knowledge, especially in the ever-changing environment, need a powerful, fast tool that should be up-to-date, low-cost, reliable, and accessible [2]. Nowadays, as a part of mHealth, teaching children and adolescents to use mobile phone educational games is one of the most time-efficient methods [3], which can be used anywhere at any time [4].

### Objectives

Consequently, the present study aimed to:

1. Design and develop a mobile-based educational game (KidFood) in pediatric nutrition.
2. Design a researcher-made questionnaire to assess the nutritional knowledge of preschool children and their parents.
3. Conduct a parallel, randomized trial (KidFood project) to compare the effect of the designed educational game with a control group.
4. Investigate the alteration in nutritional knowledge, dietary habits, dietary diversity, and anthropometric indices in 5–6 years old children after six months of intervention with a designed game.

### Hypotheses

The primary hypothesis is that KidFood will improve children's and parents' nutritional knowledge after six months of engagement compared to the control group. The secondary hypothesis is that KidFood players will adhere to more appropriate dietary habits, consume enough fruits, vegetables, and dairy products, and gain higher dietary diversity scores. The tertiary hypothesis believes that KidFood engagement can improve anthropometric indices in children 5 – 6 years old.

## Material and methods

### Project design

**Fig. 1:**
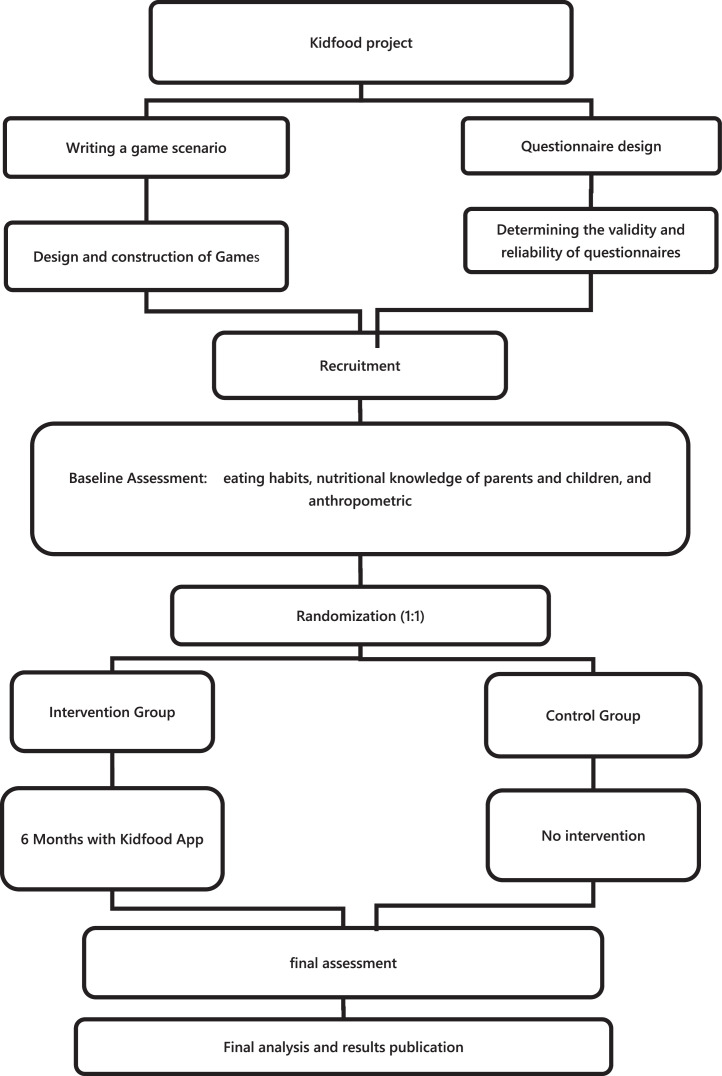
Flowchart of the Kidfood project design.

The KidFood project is a two-phase project. The KidFood mobile-based game was designed and developed in the first phase. Besides, two nutritional knowledge questionnaires were designed and validated. The second phase consists of a 6-month single-blinded parallel randomized controlled trial. The overall procedure of the project is illustrated in Fig. 1.

### Phase 1

#### Game design and development

The project's first phase consists of the nutritional education gamification named “KidFood”. The game's scenario was written by a nutritionist (MSR) and an e-learning specialist (ZK). In a first-person scenario, the character of the game (the kid) will encounter different food choices and challenges in the environments that affect children's food attitudes, including home, kindergarten, restaurant, and marketplaces. In every stage, the kid will face a character with unique nutrition-related diseases. Also, in the gameplay, a specialist nutrition character will guide the kid throughout the stages. All the guidance provided by the nutritionist character is in the form of a graphic, vocal message, and text based on the Iranian Dietary Guideline for a healthy diet.

The KidFood was developed using the “Unity game engine” by an expert team of computer sciences affiliated with the “Mahad Knowledge-based Institute of Game Development and Animation”. The gameplay and proposed scenario of KidFood consist of attractive animation, soundtrack, and story for preschool-aged children under a behavioural scientist's supervision.

The KidFood will be available at the end of the project's second phase on mobile application marketplaces. Thus, parents of participating children receive a text message (SMS) including a download link and registry key for KidFood. After installing and registering the game, one of the investigators will contact the parent and provides instructions on how to use the app.

#### Designed scenario

At first, the initial scenario of the game was set in the form of the following three training stages:

(1) Increasing nutritional knowledge by teaching food groups (based on the food pyramid), educating about main meals and healthy and unhealthy snacks, different healthy and unhealthy drinks, and based on guide lights learning enough food consumption
(2) Teaching proper nutrition to learn dietary diversity, using the experience of different spaces such as the kitchen for cooking healthy, the store for healthy food buying for teaching nutrition peers, the kindergarten, and, Restaurant for eating outside and traveling
(3) Teaching healthy eating habits and the complications of unhealthy eating habits, which is in the form of familiarization with diseases using game characters. In this phase, a nutritionist explains to the child the effect of healthy nutrition on various diseases.

#### Nutritional knowledge questionnaire

Two researcher-made questionnaires have also been designed to assess the nutritional knowledge of parents and children by (MSR, ZK, and SF) named KidFood-parent and KidFood-kid nutritional knowledge questionnaires, respectively. KidFood-kid consists of 40 and KidFood-parent consists of 35 questions based on the goals of the game. The parent questionnaire is question-based, while the child knowledge questionnaire is visual-based and uses KidFood graphics.

Before the intervention phase's initiation, an expert panel (including nine nutritionists and one health education specialist) reviewed the designed questionnaires in phase one. They evaluated the content validity using the content validity ratio (CVR) (relevance, necessity, simplicity, and clarity of items) and the content validity index (CVI). The Waltz and Bausell method [5] was used to evaluate clarity, relevance, and simplicity, and the CVR was calculated based on Lawshe [6].

Furthermore, fifty elementary school children participated to evaluate the reliability of this questionnaire using Kuder Richardson.

### Phase 2

We will evaluate the effectiveness of mHealth nutritional education using the KidFood game on preschool children aged 5 – 6 by a 6-month single-blinded parallel randomized controlled trial. This study will be conducted in the kindergartens in different urban divisions of Shiraz, Fars, Iran. The overall procedure of phase 2 is illustrated in Fig. 1. The protocol of the KidFood project was approved by the Ethics committee of Shiraz University of Medical Sciences (SUMS), Shiraz, Iran (IR.SUMS.REC.1398.332), and was registered at the Iranian Registry of Clinical Trials (IRCT.ir) under the registry code of IRCT20190810044496N1. Any changes in the methodological aspects of the project, including sample size, sampling method, randomization, and duration, also changes that affect participants' safety, will be discussed with the ethics committee of SUMS before launching the project. Parents or guardians of the children will be informed about the study and sign an informed consent form.

The present protocol article for the trial phase is based on the Standard Items: Recommendations for Interventional Trials (SPIRIT) statement [7]. The additional **file 1** is provided consists of the completed SPIRIT checklist.

#### Eligibility criteria

Preschool children aged 5 to 6 years old residing in Shiraz, who attend kindergartens located in Shiraz, will be recruited. Healthy children with no chronic mental, or physical illness who have educated parents will be known as eligible participants. Parents are asked about the lack of illness. In addition, eligible participants should have access to a mobile phone with the Android operating system. Disorders that make children ineligible to participate in the project include congenital disease, kidney and liver diseases, severe anemia, cardiovascular, lung diseases, either type 1 or type 2 diabetes, neurological, mental, and behavioural disorders such as autism and hyperactivity and attention deficit (ADHD), inflammatory gastrointestinal diseases, cancer, and movement disorders. Also, children with disorders requiring exceptional nutrition management will not enter the project. In the case of non-cooperating parents or children, the occurrence of any disease mentioned above and the loss of contentment or withdrawal of parents or children to continue cooperation with the project will lead to excluding the participant.

#### Sampling method, randomization, and blinding

Using a stratified sampling method, samples will be collected from randomly selected kindergartens in different urban areas in Shiraz, Fars, Iran. Before the baseline assessment is completed, the eligible participants are randomly allocated to either the intervention (KidFood) or control group (receive no nutritional education) at a 1:1 ratio employing computer-generated random numbers. The initial and final assessor will be blind according to the assigned group of participants. Also, the statistician analyzing the data will be blind from the allocated group, with codes assigned to groups in the data set. As the trial is an educational intervention, and the control group will not receive any training, blind participants will not be feasible. Throughout the trial, the investigation team will monitor participants to prevent information transition between groups to avoid contamination bias.

#### Sample size and power considerations

In order to provide minimum study power of 80% at the two-sided significance level (0.05), the Kid Food trial will include 120 participants (60 in each group). This sample size is determined based on KidFood-kid nutritional knowledge questionnaires.

#### Intervention

After the development of the KidFood, Kid food-parent, and Kid food -kid nutritional knowledge questionnaire, using stratified sampling, the study will be initiated. Healthy preschool children aged 5 – 6 years old of parents or guardians who have signed the informed consent form (detail of the form will be approved by the ethics committee of SUMS) will be examined for eligibility. Then, based on the previously prepared random sequence, it will be allocated to one of the study's groups. The allocated group will be concealed in a sealed envelope and will not be revealed until the baseline assessments by the investigator. A blinded assessor will record the children's frequency of food consumption, physical activity, and anthropometric indices.

After the first assessment, the participant group will be revealed, and individuals in the intervention group will receive a text message for downloading and installing the KidFood. Following the registration of the KidFood by participants, an investigator will contact the parents of the children and guide them about how to use the game and cooperate with the project. The intervention participants will play the game for one hour per day for six months (the app will not allow playing more to prevent child inactivity).

At the end of the sixth month, participants will be asked to attend kindergarten for the final assessments, which the same investigator will do. The assessor investigator will be different from the one who is aware of the participants' group.

#### Assessments

##### Demographic and health-related behaviours

At baseline, parents complete 23 items of demographic questions, including gender, date of birth, ethnicity, family status,and socioeconomic status of the family, via the occupational social class of the father and mother and the Family Affluence Scale (FAS) Questionnaire [8], as well as answer questions regarding their child's health and their education.

##### Food intake and dietary habits

Children's food intake will be assessed using a three-day food record (including two weekdays and a weekend) that will be filled by parents or other caretakers. Then, the energy and nutrient content of individuals’ diets will be analyzed using Nutritionist IV software. Children's eating habits will be defined as the number of fruits and vegetables consumed, snacks and their type, and the habit of consuming liquids such as milk, water, or sweetened beverages

##### Dietary diversity score

Kant et al.'s method [9] will be used to measure dietary diversity score (DDS). This method, will be categorized consumed food items into five food groups in the USDA Food Guide Pyramid, including grains, vegetables, fruits, meat, and dairy. The cereal group consists of seven components: refined bread, pasta, whole grain bread, cornflakes, biscuits, refined flour, and rice. Fruit will be defined by summarizing fruit and fruit juice, berries, and citrus fruits. The vegetable is a summary of potatoes, tomatoes, other starchy vegetables, legumes, yellow vegetables, green vegetables, and other vegetables. The meat group consists of red meat, chicken, fish, and eggs, and the dairy group consists of milk, yogurt, and cheese. Each group will be given a score from 0 to 2, so the maximum score will be 10. The total number of subgroups consumed by an individual is divided by the total number of subgroups and then multiplied by 2 to calculate the score for each group.

##### Growth indices

Weight is measured by trained staff and following standard methods. The weighing will be done with minimal clothing and standing in the middle of the scale (Seca model 770; Seca) with an accuracy of 100 g. Standing height is measured using a meter attached to the wall with an accuracy of 0.5 cm. Height measurement will be done when the participant's heels, shoulders, and the back of the head touch the wall with no hat or shoes.

BMI will be calculated as ([weight (kg)] / [height (m)]^2^). Then, the calculated BMI values will be adjusted for age and sex using Centers for Disease Control and Prevention charts for BMI-for-age percentiles.

##### Compliance

To ensure the adherence of children in the educational intervention group during the follow-up, the game server records the information on the entry and exit of the game daily. The recorded information will be checked daily and if there is a one-week interruption in the intervention, they will be excluded from the study.

### Statistical analysis

All statistical analyses will be performed with the Statistical Package for the Social Sciences (SPSS) (IBM SPSS Statistics for Windows, version 23.0; IBM Corp.), with a significance set at *P* < 0.05. The normality of data will be assessed using the Kolmogorov Smirnov's test. The descriptive analysis method includes reporting mean, standard deviation, median, frequency, and percentage, which will be used to describe the data depending on the type of variable (quantitative and qualitative). A paired *t*-test will be used to compare the variables before and after each group's intervention, and an Independent-sample-*t*-test will be used to compare the between-group changes.

## CRediT authorship contribution statement

**Maryamsadat Riasatian:** Conceptualization, Data curation, Formal analysis, Funding acquisition, Investigation, Methodology, Software, Writing – original draft, Writing – review & editing. **Zahra Karimian:** Conceptualization, Methodology, Validation, Visualization, Writing – review & editing. **Mohammad Ali Mohsenpour:** Writing – original draft, Writing – review & editing. **Shiva Faghih:** Project administration, Supervision, Writing – review & editing.

## Declaration of Competing Interest

The authors declare that they have no known competing financial interests or personal relationships that could have appeared to influence the work reported in this paper.

## Data Availability

Data will be made available on request.

## Appendix Supplementary materials

Image, application 1
